# Peer-Support Interventions for Self-Management in Adults Living with an Intestinal Ostomy: A Scoping Review

**DOI:** 10.3390/nursrep16090325

**Published:** 2026-09-06

**Authors:** Cátia Sofia Marques Teixeira, Amélia Filomena Oliveira Mendes Castilho, Andréa Ascenção Marques

**Affiliations:** 1 University of Coimbra, Health Sciences Research Unit: Nursing, Nursing School of the University of Coimbra, 3046-851 Coimbra, Portugal; afilomena@ese.uc.pt (A.F.O.M.C.); andreamarques23@ese.uc.pt (A.A.M.); 2 General Surgery Department, Hospital Geral, Unidade Local de Saúde de Coimbra, 3041-801 Coimbra, Portugal

**Keywords:** colostomy, ileostomy, nursing, ostomy, peer support, scoping review, self-management

## Abstract

**Background/Objectives**: This scoping review aimed to map the existing evidence on the aims, characteristics, outcomes and implementation facilitators and barriers of peer-support interventions designed to promote self-management in individuals living with an intestinal ostomy. **Methods**: A scoping review was conducted following the Joanna Briggs Institute methodology and reported according to the PRISMA Extension for Scoping Reviews (PRISMA-ScR). A systematic literature search was conducted in December 2025 in the MEDLINE (PubMed), CINAHL (EBSCOhost) and APA PsycINFO (ProQuest) databases. Quantitative, qualitative, and mixed-methods design studies written in any language were eligible for inclusion, with no restrictions on publication date, language or geographical location. Rayyan was used for duplicate removal and reference management. A total of 944 titles and abstracts were screened independently by two reviewers. Fifty-three full-text studies were assessed for eligibility. Data were extracted and synthesised descriptively and narratively. **Results**: Fourteen eligible publications were included, representing a range of study designs, including randomised controlled trials (*n* = 6), observational (*n* = 2) and quasi-experimental (*n* = 1) designs, feasibility, and economic evaluation studies. Studies were conducted primarily in the USA (*n* = 7), followed by Turkey (*n* = 3). Most peer-support interventions aimed to improve quality of life (*n* = 6). Peer-support interventions varied considerably in delivery mode, duration, facilitation, and intervention content. Interventions were delivered by peers alone (*n* = 2) or involved peers in collaboration with healthcare professionals or as part of multicomponent interventions and included face-to-face, remote, and other delivery formats, ranging from a single visit to a four-month duration. Several publications reported improvements in quality of life and self-management. Structured intervention content was reported to facilitate implementation, whereas accessibility and technological barriers were reported to limit intervention delivery. **Conclusions**: Peer-support interventions may represent a promising approach for supporting self-management among individuals living with an ostomy, particularly with several publications reporting improvements in self-management related outcomes. However, these findings should be interpreted cautiously given the heterogeneity of intervention characteristics and study designs, together with the absence of critical appraisal of included publications. Future research should identify core components of peer-support interventions and evaluate their longer-term outcomes across diverse healthcare and cultural contexts.

## 1. Introduction

A stoma is a surgically created opening that establishes a connection between the gastrointestinal tract and the abdominal wall to divert the passage of faecal matter [1]. Common indications for stoma formation include faecal diversion, bowel decompression, and protection of a gastrointestinal anastomosis. Other indications include intestinal obstruction secondary to benign or malignant tumours, perforation and peritonitis, inflammatory bowel disease, complex fistulas, mesenteric ischemia, and anorectal malformations [2,3].

Currently, no reliable global estimates are available for the number of people living with a stoma [4,5]. However, available estimates indicate that approximately 1 million people live with an ostomy in the United States [6], a similar number in China [4,7], and approximately 700,000 people in Europe [8].

Stoma-related complications, particularly leakage and peristomal skin complications, affect approximately 20 to 71% of individuals. In addition, psychological health, social functioning, and lifestyle adaptation also represent ongoing challenges for these individuals [9,10].

Self-management refers to the individual’s active participation in managing symptoms, treatment, lifestyle changes, and the physical, emotional, and social consequences of living with a chronic condition [11]. It encompasses decision-making, problem-solving, and resource utilisation, often in collaboration with healthcare professionals, to maintain or improve quality of life [11]. In the context of an intestinal ostomy, self-management includes ostomy and peristomal skin care, recognising and addressing complications, adjusting diet and physical activity, and developing coping strategies for emotional and social challenges [12,13]. Strengthening self-management skills can enhance autonomy, promote adaptation, and reduce the healthcare burden for patients and services alike [11,14]. International ostomy guidelines [9,10,15] recommend ensuring access to specialist ostomy nurses to address patients’ complex needs and to optimise the prevention and management of ostomy and ostomy-related complications [10,16]. Patient-centred nursing-care, structured training and psychological support have been shown to enhance self-efficacy, self-care knowledge, psychosocial skills, satisfaction, and overall quality of life in people with an intestinal ostomy [17,18].

Despite the benefits of specialised ostomy nursing care, many individuals continue to experience a substantial care burden and report unmet supportive care needs following ostomy surgery. Common unmet needs include feeling insufficiently prepared for self-management, inadequate opportunities for hands-on training, and difficulties managing fatigue, sleep disturbances, and dietary changes, as well as limited support or referral for concerns related to sexuality and intimate relationships [19,20]. These challenges may be further exacerbated by increasingly shorter postoperative hospital stays and unequal access to specialist stoma nursing services after discharge. Together, these gaps highlight the need for integrated models of care that extend beyond the acute care setting, incorporating comprehensive preoperative education, structured post-discharge follow-up, and complementary psychosocial interventions to support long-term adaptation and self-management [19,21]. Peer support has therefore emerged as a complementary strategy to promote self-management among people living with an intestinal ostomy. Through the sharing of lived experience, peers provide emotional, informational, and practical support that can strengthen self-efficacy, facilitate adaptation, and foster meaningful social connectedness [22,23].

Peer support has been successfully implemented across a range of chronic conditions including mental health, cancer, diabetes, HIV, arthritis, atrial fibrillation, chronic pain, inflammatory bowel disease, multiple sclerosis, peripartum cardiomyopathy, and stroke, [23,24,25] and evidence is emerging in ostomy patients [26,27]. Peer support may occur through a multifaceted undertaking, encompassing various forms and settings, in group or individual sessions, via face-to-face or remote delivery assistance [23,24,28,29]. Reported outcomes of peer-support interventions (PSIs) have included improvements in quality of life, social participation, self-efficacy, and health-directed activity [24,25,27,29,30]. Furthermore, ostomy patients have described renewed hope and confidence in resuming everyday life, and increased social connections [26,31] after attending a support group.

Bandura’s Social Cognitive Theory provides one possible explanation for how peer support may promote self-management behaviours. According to this theory, individuals learn through observing others, particularly credible role models with relevant lived experience. In the context of intestinal ostomy care, peers may strengthen self-efficacy by sharing practical knowledge, demonstrating self-care strategies, and providing encouragement, thereby supporting adaptation and confidence in managing life with an ostomy [32,33].

These considerations underline the growing interest in peer support as a valuable adjunct to clinical care, while emphasising the importance of understanding PSI characteristics to inform their integration into nursing practice.

Previous evidence syntheses have examined peer support across physical health conditions and healthcare settings [34,35], while systematic reviews have focused on specific outcomes among people with cancer and inflammatory bowel disease [28,36]. In ostomy care, evidence synthesis has primarily addressed the effectiveness of nursing [37] and of self-management interventions [38]. However, evidence on peer support for people living with an intestinal ostomy remains fragmented, with substantial variation in intervention objectives, delivery modalities, peer preparation, outcomes, and implementation strategies. Given this breadth and heterogeneity, a scoping review was considered appropriate to map the characteristics, outcomes, and implementation features of existing PSIs rather than to determine their comparative effectiveness. To our knowledge, no scoping review has comprehensively mapped these aspects. Therefore, the aim of this scoping review was to map the existing evidence on PSIs for self-management promotion in individuals living with an intestinal ostomy, specifically focused on the aims, characteristics, outcomes and facilitators and barriers to intervention implementation.

## 2. Materials and Methods

A scoping review was conducted in accordance with the Joanna Briggs Institute (JBI) methodology [39], and reported following the PRISMA-ScR [40] guidelines. Database searches were completed in December 2025. A protocol for this review was prospectively registered on the Open Science Framework on 6 February 2025, before study screening commenced, and is available at: https://doi.org/10.17605/OSF.IO/2FY3G (accessed on 30 August 2026).

The inclusion criteria of this scoping review were based on the “PCC” mnemonic: Population, Concept, and Context. Accordingly, this review considered all publications that focused on adults (≥18 years) living with a temporary or permanent intestinal ostomy created for malignant or benign conditions. For this review, the potential to regain autonomy referred to the capacity to progressively develop or regain independence in stoma self-care and daily activities, including managing the stoma and appliance, recognising and responding to problems, and making informed decisions about their care. Studies involving mixed ostomy populations, including urostomies, were eligible when individuals with an ileostomy and/or colostomy represented at least 50% of the sample (Population). Publications evaluating interventions in which peer support constituted either the primary intervention or an explicit component of a multicomponent intervention were eligible. To be included, peer support had to be intentionally incorporated as a planned intervention. For the purpose of this review, peer support was defined as the provision of emotional, appraisal, informational, or practical support by individuals who share relevant lived experience with the target population. In this review, a peer was defined as an individual with lived experience of living with an intestinal ostomy, who intentionally participated in a planned peer-support intervention to support another person facing similar challenges through the sharing of experiential knowledge. Peers were not required to hold a professional healthcare qualification or formal clinical training in ostomy care. Where applicable, peer preparation or training in ostomy care, communication, peer-support skills, or intervention-specific procedures was considered an intervention characteristic and extracted accordingly. Informal support provided incidentally by family members, friends, or individuals without an intentional peer-support role was not considered peer support [23,41,42]. Peer support was distinguished from related concepts according to the intentional role and lived experience of the peer within the intervention. Patient education, psychosocial support, self-management programmes, patient navigation, and technology-assisted care were not considered peer support in themselves; these approaches were eligible only when an individual with relevant lived experience of an intestinal ostomy had an explicit peer-support role, such as providing experiential knowledge, emotional or informational support, or facilitating peer interaction. Similarly, telehealth, mobile applications, and remote monitoring were considered delivery modalities rather than peer support unless they incorporated an explicit peer-support component. Publications were eligible when peer support was an intentional and planned component of the intervention, whether as a primary intervention or as part of a multicomponent intervention. Conversely, publications in which peers were involved only incidentally, without an explicit peer-support role, were excluded (Concept). Included publications encompassed a wide range of settings, including hospitals, outpatient clinics, acute care, and analogous contexts (Context).

Quantitative, qualitative, and mixed-methods design publications written in any language, with no chronological, language or geographical constraints, were included. Reviews, case reports, editorials, commentaries, letters, conference abstracts, and publications that did not evaluate a peer-support intervention were excluded.

### 2.1. Search Strategy

An initial limited search of MEDLINE (PubMed) and CINAHL (EBSCOhost) was undertaken to identify relevant articles and refine the search strategy. Keywords and index terms identified from these records informed the development of the final search strategies, which were adapted for each database. The text words contained in the title and abstract, and the index terms used to describe relevant articles, were used to develop a full search strategy. Later, a search using all identified keywords and index terms was undertaken across MEDLINE (PubMed) and CINAHL (EBSCOhost) and APA PsycINFO (ProQuest) databases. Finally, a comprehensive search of the reference list of all identified articles was undertaken to locate additional studies.

The final search strategy was executed in MEDLINE (PubMed), CINAHL (EBSCOhost), and APA PsycINFO (ProQuest) on 31 December 2025 (Appendix A). Backward and forward citation searching of relevant publications was undertaken to identify potentially eligible studies not retrieved through database searching. Grey literature searches were conducted on 31 December 2025 by screening the websites and organisational repositories from the Portuguese Association of Stoma Care Nursing, the Registered Nurses’ Association of Ontario, the Wound, Ostomy and Continence Nursing, the European Council of Enterostomal Therapists and the World Council of Enterostomal Therapists.

### 2.2. Study Selection

Following completion of the searches, all identified records were uploaded into Rayyan available online: https://www.rayyan.ai/ (accessed on 5 January 2026) and duplicates were removed. Prior to formal screening, the two reviewers (CT and AM) independently piloted the eligibility criteria using a sample of records to ensure clarity and consistent application. Any discrepancies identified during pilot screening were discussed and the eligibility criteria clarified before formal screening.

Thereafter, titles and abstracts were screened by two independent reviewers (CT and AM) for assessment considering the inclusion criteria for the review. Potentially eligible reports were retrieved in full text and independently assessed by the same two reviewers.

The full text of selected citations was assessed by two independent reviewers (CT and AM). Any disagreements between the reviewers at each stage of the selection process were resolved through discussion and, when consensus could not be reached, consultation with a third reviewer (AC).

### 2.3. Data Extraction

Data were extracted using a structured data-charting form developed by the review team and piloted on a sample of included publications. The form was designed to capture information relevant to the review question, including study characteristics, participant characteristics, intervention aims and characteristics, timing, delivery mode and duration, peer and healthcare-professional involvement, intervention content and resources, reported outcomes, and implementation facilitators and barriers.

Facilitators and barriers were charted according to how they were reported in the included publications in relation to intervention implementation, participation, delivery, or acceptability. Data were initially extracted according to the terminology and interpretation provided by the original study authors and were subsequently grouped according to conceptual similarity while preserving their original meaning and contextual interpretation. No predefined classification framework was imposed. Facilitators and barriers were classified as implementation determinants when the included studies described them as factors that supported or hindered intervention participation, delivery, acceptability, accessibility, or sustainability. Intervention characteristics were not classified as facilitators or barriers solely on the basis of being components of the intervention; rather, their reported influence on implementation was considered. Outcomes resulting from the intervention, such as clinical, psychosocial, or economic outcomes, were classified separately and were not considered implementation determinants.

Data were independently extracted from the included publications by two reviewers (CT and AM) using a standardised data extraction tool developed by the review team. The tool was piloted on a sample of included publications to ensure consistency between reviewers before full data extraction commenced.

### 2.4. Data Synthesis

Extracted data were synthesised descriptively and narratively in accordance with the objectives of the review. Study characteristics, including publication year, country, study design, and intervention delivery format, were summarised using frequency counts and presented in tables. Intervention characteristics, aims, reported outcomes related to ostomy self-management, and factors influencing programme implementation were compared across included publications and mapped to identify patterns, similarities, and gaps in the available evidence.

Reported outcomes were grouped into broader categories when they represented conceptually similar or related constructs. Facilitators and barriers to intervention implementation were initially extracted according to the terminology and interpretation provided by the original study authors and subsequently grouped according to conceptual similarity while preserving their original meaning and contextual interpretation. No predefined classification framework was imposed. This iterative descriptive process was used to organise and map the available evidence rather than to generate new analytical themes.

For frequency-based reporting, frequencies represent the number of included publications reporting a given study or intervention characteristic, outcome category, facilitator, or barrier. For each specific category, each publication was counted only once, regardless of the number of findings reported within that category. Frequencies were used descriptively to identify patterns in the mapped literature and should not be interpreted as measures of effect, prevalence, or strength of evidence.

Multiple publications arising from the same intervention were eligible for inclusion when they addressed distinct research objectives, populations, study designs, or dimensions of the intervention relevant to the review question. These publications were retained as separate reports but were identified as originating from the same overarching intervention, thereby ensuring transparency and avoiding their interpretation as independent interventions.

Accordingly, frequency counts were performed at the publication level and do not necessarily represent independent interventions or participant samples. Any disagreements between reviewers were resolved through discussion with a third reviewer (AC). Where necessary, corresponding authors were contacted to clarify missing or incomplete information.

In accordance with JBI guidance for scoping reviews, no critical appraisal of individual sources of evidence was performed, as the aim was to map the existing literature rather than assess study quality.

## 3. Results

### 3.1. Study Characteristics

**Figure 1:**
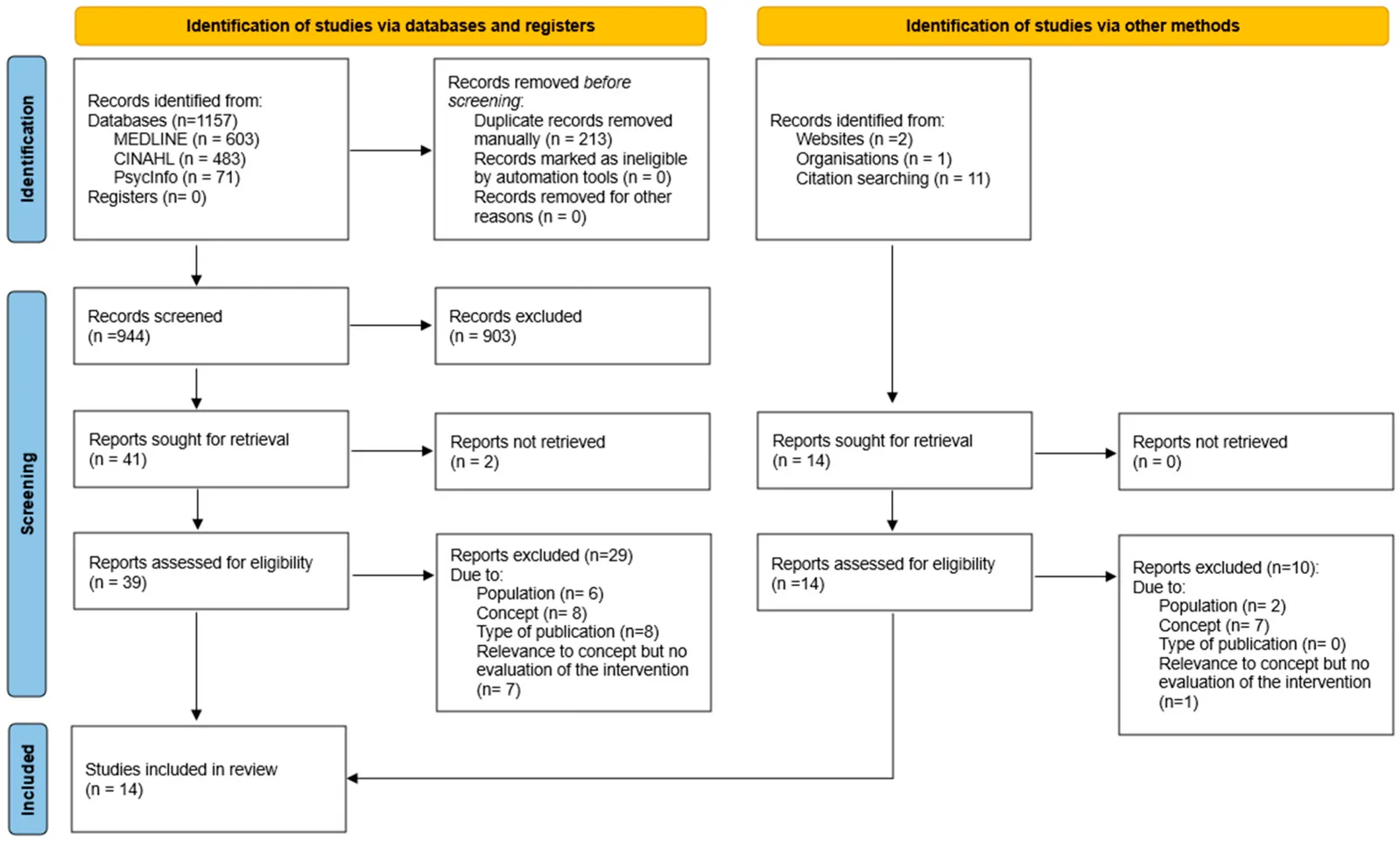
PRISMA 2020 flow diagram of study identification, screening, and inclusion.

A total of 1157 articles were retrieved from the databases. The results of the study selection process are presented in a PRISMA flow diagram (Figure 1) [43].

The 14 included publications were published from 2012 [44] to November 2025 [45]. Most of the publications were undertaken in North American countries [46,47,48,49,50,51,52] (*n* = 7), followed by Asian [44,45,53,54,55] (*n* = 5) and European [56,57] (*n* = 2). All included publications were in English. Randomised controlled trials were the predominant study design [49,50,52,55,56,57], although observational [46,53] studies, quasi-experimental [54], feasibility pilot study trial [48],and economic analyses [47] studies were also identified. Except for one [46], the majority [45,47,48,49,50,51,52,53,54,57] of publications identified cancer disease as the main reason for ostomy creation. Six included publications were related to the Ostomy Self-Management Telehealth intervention. Although originating from the same overarching intervention, these publications addressed distinct aspects of its development, implementation, and evaluation, including pilot testing and feasibility, intervention design and theoretical foundations, economic evaluation, technological aspects of telehealth delivery, experimental evaluation, and adaptation to rural populations. These publications were therefore retained separately while being identified as linked to the same intervention. The summary of characteristics of included publications in the scoping review is described in Table 1.

### 3.2. Intervention Aims

Although PSIs for ostomy patients aimed at different self-management-related objectives, across publications, interventions primarily aimed to improve quality of life [47,48,49,50,51,55], to enhance ostomy self-efficacy [44,52,57], to assist patients to adjust to ostomies [47,48,49,50], and to reduce healthcare service utilisation [46,50,57]. Less frequently, interventions sought to encourage preventative health behaviours [46], promoting patient activation [52] and reducing reliance on caregivers [57].

### 3.3. Intervention Characteristics

The features of ostomy PSIs for self-management of people with an intestinal ostomy, including delivery and administration, format and duration, content developers, content and resources, techniques and strategies, leading, and participant:peer ratio are outlined in Table 2.

Overall, PSIs were predominantly delivered face-to-face, although several publications incorporated remote telehealth approaches, including videoconferencing, mobile health platforms and hybrid delivery models. Interventions were most commonly initiated during the postoperative period, with a small number beginning preoperatively. Intervention duration varied considerably, ranging from a single session or day to four months, although most extended over several weeks or months through weekly sessions lasting approximately 60–120 min; a few interventions provided ongoing or continuous support. Intervention content was primarily developed by ostomy nurses, frequently in collaboration with research nurses and surgeons, while direct involvement of patients or peer supporters in intervention development was less commonly reported. The interventions addressed both technical (e.g., ostomy care, complication prevention and healthy lifestyle) and psychosocial aspects of self-management, using a range of educational resources including printed materials, audiovisual tools, and digital platforms. Most interventions incorporated trained peers, often matched to participants according to characteristics such as age or gender, and were commonly co-facilitated by peers and healthcare professionals. Educational strategies typically combined structured teaching with group discussion, experience sharing, role-playing, and problem-solving approaches. Delivery formats ranged from individual peer-to-peer support to small group interventions, with participant-to-peer ratios varying substantially across publications.

#### 3.3.1. Delivery and Administration

Different formats of delivery of PSIs were identified in the included literature. Most publications addressed face-to-face [44,45,47,48,53,54,56] peer-support interaction. However, remote [46,49,50,51,52,57] telehealth approaches were explored via video conferencing [49,50,51,52] or through mobile healthcare applications [46,57]. Recently, a hybrid [55] format combined remote consultations and face-to-face meetings.

#### 3.3.2. Format and Duration

Intervention durations ranged from a single session via a visit [56] or a one-day [53] session, to interventions lasting four months [45], with most interventions extending over several weeks or months [44,45,46,47,48,49,50,51,52,54,55,57]. Weekly sessions [44,49,52,54] were the most commonly reported delivery schedule, although continuous [46,57] support models were also identified. Individual sessions typically lasted between 60 min [47,48,51] and 90 to 120 min [44,45,49,50,52,54,55], with some extending beyond two hours [53]. Most interventions were initiated predominantly during the post-operative phase [44,45,49,50,51,52,53,54,55,56], although a small number [46,47,48,57] commenced preoperatively.

#### 3.3.3. Content Developers

The development and consultation of PSIs content for ostomy self-management was primarily undertaken by ostomy nurses [44,47,48,49,50,51,52,53,54,55], frequently with contributions from research nurses [44,45,47,48,49,50,51,52,54], surgeons [47,48,49,50,51,52,53] and other health professionals [53,55]. Only a minority reported patient and public involvement [45,55,57], including contributions from individuals living with an ostomy or peer supporters, during intervention content development.

#### 3.3.4. Content and Resources

The content of PSIs was fully described in most publications, although two publications provided only limited information on intervention content [46,55]. Overall, interventions addressed a broad range of self-management domains extending beyond technical ostomy care. Common topics included ostomy care and daily life [44,45,47,48,49,50,51,52,53,54,57], healthy lifestyle [45,47,48,49,50,51,52], complication prevention [44,53] social well-being and body image [47,48,49,50,51,52] and social interaction [45,53,54,56]. Several interventions also incorporated information on ostomy output [46,57], device management and skin condition [46], clinical pathway [57] and patient associations and healthcare resources [57].

A variety of educational resources supported intervention delivery, reflecting the diversity of intervention formats. These included printed educational materials (e.g., self-management handbook [44], brochures [53,55], workbook [49,50,51,52], and resource list [49,50,51,52]), audiovisual resources (e.g., video [45,53,55,57], slide presentations [51,54], DVD [47,48], diagrams [49,50,51,52], photos [49,50,51,52] and close-ups views [49,50,51,52] and reminder calls [35,37,38,44]), and digital technologies such as video conference platforms [49,50,51,52], mobile healthcare application [46,57], peer chat functions [57], WeChat [55], and electronic personal notifications reminders [46,57]. Some interventions also incorporated practical resources, including ostomy model and materials [54] and hands-on training and demonstrations [47,48] to reinforce self-management skills.

Human resources were a key component of intervention delivery. Most interventions involved trained peers [44,46,47,48,49,50,51,52,55,56], gender-matched peers [47,48,49,50,51,52] and gender- and age-matched peers [56] to facilitate rapport and shared experiences. Additional strategies included supplemental peer contact [48], apeer-selection feature [57] within digital platforms and home visits [55].

#### 3.3.5. Techniques and Strategies

Among the numerous techniques used on PSIs, the most common educational strategies included structured content [44,47,48,49,50,51,52,53,54], group education [44,45,47,48,49,50,51,52,53,54], discussion [45,47,48,49,50,51,52,54] and role-playing [47,48,49,50,51,52] and personalised educational content [45,55].

Most frequently applied strategies encompassed experience-sharing [44,45,46,47,48,49,50,51,52,53,54,55,56,57], adults teaching principles [47,48,49,50,51,52], problem-oriented approaches [47,48,49,50,51,52], distance learning teaching [49,50,51,52] and digital health education [46,57].

#### 3.3.6. Leading and Participant:Peer Ratio

Intervention facilitation varied across interventions. Two interventions were peer-led [44,56], two were peer-assisted [46,57], and seven involved peers in collaboration with healthcare professionals, predominantly ostomy nurses [47,48,49,50,51,52,55]. Three interventions were professionally led without an explicitly identified peer-facilitation role [45,53,54]. Thus, although trained peers were incorporated into most interventions, their roles ranged from primary facilitation to complementary support.

Similar patient-to-peer distributions were addressed on one-to-one interaction [56,57] and on more numerous groups ranging between four to twelve [45,49,50,52,54] participants. Non-equal distributions of four to fourteen patients to two peers’ [44,47,48,51,55] interactions were also provided.

### 3.4. Intervention Outcomes

#### 3.4.1. Psychosocial Outcomes

Psychosocial outcomes were the most reported across the included publications. Improvements in quality of life [45,47,48,49,51,53,55,57] were the most consistently reported outcome following participation in PSIs. Several publications also described enhanced psychosocial adjustment to ostomy [44,45,53,54], improved social well-being [47,48,51,52] and greater social reintegration, including a higher likelihood of returning to full-time employment and a lower probability of claiming disability six months after the intervention reported [50].

One publication reported higher scores for coping processes, health beliefs, and acceptance of health status among participants receiving the intervention compared with those receiving standard care [56]. Reductions in anxiety and depression were reported in some studies, including at the six-month follow-up [45,48,49]. Improvements in role-emotional and mental health measures were also reported among specific participant groups, including married individuals and rural cancer survivors [53]. A mobile health intervention reported improvements in stoma-related quality of life and psychological adaptation during the early postoperative period [57].

#### 3.4.2. Self-Management Outcomes

Across the included publications, a range of self-management-related outcomes were reported following peer-support interventions. Several publications evaluating structured education and peer-support approaches reported improvements in self-efficacy and patient activation [44,48,49]. One publication reported improvements in ostomy-related knowledge and self-care abilities following an educational intervention [44], while another reported improvements in self-care skills, self-responsibility, and health-related concepts [55]. Higher attendance at training sessions was reported to be associated with greater improvements in some self-efficacy domains, including obtaining help and managing symptoms [49]. Improvements in patient activation, measured using the Patient Activation Measure, were reported in pilot research [48]; however, findings from a larger trial suggested that changes in self-efficacy varied across participant subgroups, with greater improvements reported in some domains among urological cancer survivors [49].

#### 3.4.3. Healthcare Utilisation and Clinical Outcomes

Economic outcomes were also reported. One economic evaluation indicated that face-to-face peer-support intervention delivery was reasonably cost-effective [47]. A telehealth-delivered intervention was associated with lower direct out-of-pocket healthcare and travel costs and fewer in-person consultations [50].

Remote delivery was also reported to facilitate access to peer support among participants living in geographically dispersed areas [52]. Increased satisfaction with ostomy care was reported following participation in peer-support interventions [48,55].

Clinical outcomes included the occurrence and severity of peristomal and stoma-related complications. One publication reported fewer complications, including stoma bleeding, oedema, and peristomal dermatitis, following an intervention combining visual and peer education [55]. Another reported earlier recognition and lower severity of peristomal complications among participants receiving peer-support interventions [45]. A remote monitoring intervention reported 30-day readmission and emergency department visit rates of 15.1% and 6.1%, respectively, which were lower than the national benchmarks reported by the authors for individuals with a new ostomy (24.7% and 17.7%, respectively) [46]. These comparisons should be interpreted in the context of the original publication design and comparator or benchmark used.

Overall, the included publications reported outcomes related to healthcare utilisation, complications, costs, access, and satisfaction, but findings were heterogeneous and were assessed using different approaches and follow-up periods. The reported findings therefore map the range of healthcare and clinical outcomes investigated in peer-support interventions rather than establishing their effectiveness. Detailed outcome data, including measurement approaches and reported findings, are presented in Supplementary Table S1.

### 3.5. Implementation Facilitators and Barriers

Reported facilitators and barriers to the implementation of PSIs are clustered into several common domains (Table 3).

#### 3.5.1. Facilitators

Several publications identified factors supporting the implementation of PSIs for ostomy self-management. Experiential knowledge-sharing [44,45] and problem-solving learning strategies [54] supported communication, learning, and self-management. Peer-led education, either independently or in partnership with healthcare professionals, was reported to facilitate knowledge transfer and reduce communication barriers [44,53,54,55,57]. Tailoring educational content to participants’ needs and preferences [45,57] also supported engagement and participation.

Active patient involvement in meeting organisation, peer interaction, and intervention design was reported to facilitate engagement and participation [53,55,57]. Incorporating patients’ identified needs and preferred intervention functionalities [53,55,57], together with collaboration between ostomy nurses, peers, and patient associations [44,55,57], supported intervention delivery and acceptability.

Remote delivery through video conferencing [49,50,51,52], mobile healthcare applications [46,57] and WeChat [55] was reported to facilitate participation and access to peer support by reducing geographical and travel-related barriers, particularly for individuals living in rural or geographically dispersed areas.

#### 3.5.2. Barriers

Diverse barriers to the implementation of PSIs for ostomy self-management were identified. Cultural beliefs, stigma, and societal taboos surrounding sensitive topics, particularly sexual health, were reported to limit patient participation [44].

For face-to-face interventions, travel distance [47,48,51], transportation difficulties [47,48], physical limitations following surgery [47,48,49], and financial constraints [48,49] were reported to restrict participation and access to peer support.

Although digital delivery facilitated accessibility, technical and organisational challenges were also reported. These included the development and maintenance costs of mobile health applications, delayed or inadequate responses to technical problems, and the need for organisational and financial resources to maintain digital platforms and technological infrastructure [57].

## 4. Discussion

This scoping review mapped the available evidence on PSIs for adults living with an intestinal ostomy and demonstrated that these interventions are increasingly incorporated into ostomy care across different healthcare settings. Overall, the included publications reported improvements in several psychosocial outcomes, particularly quality of life, self-efficacy, psychological adjustment, knowledge, patient activation, and adaptation to life with an ostomy. Diverse publications also reported benefits in healthcare-related outcomes, including patient satisfaction, postoperative recovery, and cost-effectiveness [46,50,56,57]. However, considerable heterogeneity was identified in intervention design, delivery, leadership, timing, duration, and outcome assessment, limiting comparisons across publications and precluding conclusions regarding the optimal model of peer support. One of the most important findings of this review is the substantial diversity in how PSIs are conceptualised and implemented. Considerable variation was observed in intervention duration, timing, delivery mode, educational content, leadership, peer preparation, and participant matching, in accordance with previous findings from other investigations [22,23,25,30,58,59] of PSIs across chronic conditions. While this heterogeneity reflects adaptation to different healthcare systems, patient populations, and available resources, it also highlights the absence of consensus regarding the essential components of effective ostomy PSIs.

Quality of life emerged as the most frequently evaluated outcome, reflecting the multidimensional impact of intestinal ostomy on physical, psychological, social, and functional well-being. Similar findings have been reported in PSIs developed for individuals with bladder cancer [27], breast cancer [29], diabetes mellitus [25,58] or brain injury, cerebral palsy, and spina bifida [30], suggesting that quality of life represents a core outcome across long-term conditions. Improvements in self-efficacy, patient activation, knowledge, social adaptation, and reductions in anxiety and depression were also commonly reported, reinforcing the contribution of peer support to self-management and psychosocial adjustment [22,23,25,27,29]. The review further identified positive healthcare-related outcomes, including improved postoperative recovery and greater patient satisfaction.

Favourable economic observations were also reported, with one study reporting reasonable cost-effectiveness and another reporting reduced travel costs. These findings should be interpreted cautiously given the limited economic evidence available. Although one study reported higher 30-day readmission rates, these were largely attributed to surgery-related factors rather than intervention failure. Previous research [20,21,23] similarly demonstrates that ostomy-related complications contribute substantially to emergency department visits, hospital readmissions, and healthcare costs, highlighting the potential value of interventions that strengthen patient education and self-management.

Educational content frequently extended beyond technical ostomy management to include psychosocial adjustment, lifestyle adaptation, coping strategies, emotional support, and experience sharing. Most interventions were developed or coordinated by ostomy nurses, often in collaboration with multidisciplinary teams, peers, or patients. These findings align with the 2020 International Ostomy Guidelines [15], which advocate for holistic education throughout the perioperative pathway and recognise the importance of addressing both physical and psychosocial needs. The methodology of the third edition of the International Ostomy Guidelines [60] similarly aims to promote self-management and health-related quality of life across care settings and countries.

The transferability of these findings should also be considered in relation to the geographical and healthcare contexts in which the included publications were conducted. Most evidence originated from the USA, China, and Turkey, where differences in healthcare-system organisation, access to specialist ostomy care, and the availability and role of stoma nurses may influence the implementation and uptake of PSIs. Cultural perceptions of ostomy, preferences for seeking support, and attitudes towards sharing personal experiences with peers may also shape engagement with these interventions. Therefore, intervention components and outcomes observed in these settings may not be directly transferable to healthcare systems with different professional roles, resources, or models of ostomy care. As these contextual factors were not consistently reported across the included publications, their influence could not be systematically examined. Future research should therefore evaluate the implementation and effectiveness of PSIs across diverse healthcare and cultural contexts.

The review also demonstrated increasing adoption of digital and hybrid delivery models, including video conferencing and mobile health applications. These approaches may overcome geographical, mobility, and body image barriers that frequently limit participation in conventional face-to-face support. Previous evidence [61] has similarly shown that telehealth interventions improve self-care, knowledge, self-efficacy, quality of life, adaptation, and patient satisfaction. Nevertheless, successful implementation depends on adequate technological infrastructure, digital literacy, and appropriate technical support [24,61,62].

Another notable finding was the predominance of postoperative interventions. Although peer support was primarily introduced during rehabilitation, international guidelines [15] recommend that education and psychosocial preparation begin before surgery and continue throughout the perioperative trajectory. Earlier integration of peer support may better prepare patients and families for ostomy creation and facilitate adaptation from the outset [15,63].

The findings reinforce the central role of ostomy nurses in the development, coordination, and delivery of PSIs. Beyond providing clinical care, ostomy nurses are well positioned to facilitate self-management, identify patients’ educational and psychosocial needs, prepare and supervise peer supporters, and ensure that peer-delivered information complements evidence-based clinical practice.

The review also highlights the importance of adopting a person-centred approach when implementing PSIs. Educational content should be tailored to individual needs, concerns, readiness, and preferences [17,23,30,64,65,66], while peer matching based on characteristics such as age, gender, lifestyle, or lived experiences [12,22,28,59,65] may enhance intervention relevance and participant engagement. Other studies have indicated the use of trained peers [12,27,30,58] with training covering health-promoting behaviours, facilitation, group management, problem solving, empathy, and communication. A study [29] warned that lack of training may result in incomplete or biased information. Integrating peer support alongside routine nursing care throughout the perioperative pathway may contribute to improved adjustment, greater confidence in self-care, and enhanced quality of life. Successful implementation of PSIs depends on addressing both organisational and patient-related factors. Facilitators identified across the evidence included collaboration between ostomy nurses and trained peer supporters, structured peer preparation, individualised education, flexible delivery formats, and opportunities for experience sharing and emotional support. Hybrid models combining face-to-face and remote interactions [24,25,61,67] appear particularly promising, offering greater accessibility while maintaining interpersonal connection. Several implementation barriers were also identified. Cultural stigma surrounding ostomy, geographical distance, transportation difficulties, limited technological infrastructure, insufficient digital literacy, and healthcare resource constraints may reduce intervention accessibility. Furthermore, culturally and linguistically diverse populations may require peer matching that considers language, cultural background, and lived experiences [29,65,68]. These findings suggest that successful implementation requires flexibility, organisational support, and adaptation to local healthcare contexts while preserving core intervention components [24].

Despite encouraging findings, the current evidence remains characterised by considerable methodological and intervention heterogeneity. Future research should prioritise the development of consensus regarding the core components of effective PSIs, including intervention content, peer selection and training, intervention intensity, delivery methods, and outcome measurement. Adoption of standardised reporting frameworks would improve intervention reproducibility and facilitate evidence synthesis.

Further multicentre, adequately powered randomised controlled trials with longer follow-up are needed to evaluate the sustainability of intervention effects and identify the most effective intervention models. Future studies should also evaluate implementation outcomes, cost-effectiveness, digital delivery models, caregiver involvement, and strategies for integrating peer support across the entire perioperative care pathway. Greater attention should also be given to culturally diverse populations and underserved settings to improve equity in access to peer support.

### Strengths and Limitations

This review has several strengths. The protocol was prospectively registered, enhancing methodological transparency and reducing the risk of reporting bias. Comprehensive searches were conducted across multiple databases without language or date restrictions, enabling the inclusion of evidence from diverse healthcare settings. Furthermore, adherence to PRISMA-ScR reporting recommendations strengthened the transparency and reproducibility of the review process.

The findings of this review should be interpreted considering some limitations related to the search strategy. Requiring publications to explicitly address ostomy, peer support, and self-management may have reduced the sensitivity of the search for studies in which self-management was described using related concepts or alternative terminology. In addition, the search was limited to three databases, and relevant studies indexed exclusively in other databases may therefore not have been identified. Although the database search was complemented by targeted grey literature searching through professional organisations specifically related to stoma care, the characteristics of these websites and repositories did not allow the use of structured search dates or search terms.

A further challenge concerned the conceptual and terminological variability surrounding peer support. Although an extensive search strategy was developed through an iterative process that included examination of peer-support-related literature, identification and testing of alternative terminology, and refinement of search terms, differences in how peer support is defined, labelled, and operationalised across studies may have resulted in relevant evidence being missed. Conversely, the broad conceptualisation of peer support may have increased heterogeneity among the included interventions. These limitations should be considered when interpreting the mapped characteristics and reported outcomes of PSIs for people living with an intestinal ostomy. The review deliberately focused on peer-based interventions and therefore excluded interventions centred primarily on family support, although family involvement is recognised as important in ostomy rehabilitation. In publications evaluating multicomponent interventions, the specific contribution of peer support could not always be disentangled from that of the other intervention components. Consequently, the reported benefits of these interventions cannot be attributed exclusively to peer support.

In accordance with JBI guidance for scoping reviews, no critical appraisal of the methodological quality of individual sources of evidence was undertaken. Therefore, the findings should not be interpreted as an assessment of the methodological rigour, certainty, or strength of the evidence. The findings and conclusions should also be interpreted in light of the methodological characteristics of the included publications. Consequently, although the available evidence frequently supports the potential benefits of PSIs, stronger evidence from rigorously designed multicentre studies is required to establish best practice.

## 5. Conclusions

This scoping review provides the first comprehensive mapping of PSIs designed to promote self-management among people living with an intestinal ostomy. The available literature is heterogeneous in terms of intervention aims, delivery models, duration, content, and outcomes assessed. Across the included publications, potential benefits of peer support were reported in relation to self-management, psychosocial adjustment, quality of life, and selected healthcare outcomes; however, these findings should be interpreted cautiously given the heterogeneity of the evidence, the absence of critical appraisal, and the inclusion of multicomponent interventions in which the specific contribution of peer support could not always be isolated.

The review also identified facilitators and barriers relevant to the design and implementation of PSIs, including peer preparation and training, involvement of ostomy nurses, flexible delivery formats, and attention to cultural and technological accessibility. These findings provide a descriptive basis for considering how peer support may be incorporated into person-centred ostomy care, while highlighting important gaps in the current evidence. Future research should further define the core components and mechanisms of PSIs, use more consistent outcome measures, and employ robust study designs to evaluate their effectiveness and implementation over time and across diverse healthcare settings and cultural contexts.

## Figures and Tables

**Table 1:** Summary of characteristics of included publications.

Study (Year)	Country	Study Design	Sample *n*	Age (Years)	Sex	Setting	Comparator	Follow Up	Reasons for Ostomy	Ostomy Type	Ostomy Duration
Cheng et al. [44] (2012)	China	PPC	81 (plus 11 expert patients)	18 to 75	56 (69.1%) Male, 25 (30.9%) Female	Stoma clinic in provincial hospital, Nanjing	Single group (pre vs. post)	4 weeks post-programme	NA	Colostomy	Permanent
Altuntas et al. [53] (2012)	Turkey	Observational	72	56.8 ± 13.6	44 (61.1%) Male, 28 (38.9%) Female	Kartal Education and Research Hospital, Istanbul	No control; pre- vs. post-education	3 months post-intervention.	Cancer (*n* = 68, 94.4%) Non-cancer (*n* = 4, 5.6%)	Colostomy Ileostomy Urostomy	Permanent Temporary
Karabulut et al. [54] (2014)	Turkey	QE	50 (23 exp, 27 control)	Exp: 55.5 ± 9.9; Control: 53.8 ± 10.0	Exp: 12 (52.2%) Male; Control: 18 (66.7%) Male	Stomatherapy Unit, Gazi University, Ankara	Routine care	Baseline, 6 weeks, and 10 weeks	Cancer (*n* = 22, 95.7%) Non-cancer (*n* = 1, 4.3%)	Colostomy Ileostomy	Permanent Temporary
Krouse et al. * [48] (2016)	USA	FPS	38	60–82 (Mean: 71.3)	28 (73.7%) Male, 10 (26.3%) Female	Community setting (Tucson, Arizona)	No comparator	Baseline, post-intervention (session 5), and 6-month follow-up.	Cancer (*n* = 38, 100%)	Colostomy Ileostomy Urostomy	NA
Hornbrook et al. * [47] (2018)	USA	EARCT	35	NA	Male and female (gender-matched buddies)	University of Arizona/Tucson metropolitan area	NA	Analysis of incremental cost: Baseline to end of 8-week intervention.	Cancer (*n* = 35, 100%)	Colostomy Ileostomy Urostomy	NA
Sun et al. * [51] (2018)	USA	RCT Study design	NA (IG: *n* = 162)	Adults (≥21)	Both (stratified randomization)	Home-based (telehealth)	Usual care	Baseline, immediate post-intervention (session 4), and 7 months.	Cancer (*n* = 162, 100%)	Colostomy Ileostomy Urostomy	Permanent Temporary
Fearn et al. [46] (2020)	USA	Observational	66	19–75 (Mean: 43.1)	30 (45.5%) Male, 36 (54.5%) Female	19 institutions; remote monitoring via smart platform	Historical national dataset	30-day after ostomy surgery	Cancer (*n* = 21, 31.8%) Non-cancer (*n* = 35, 53%) Unknown (*n* = 10, 15.1%)	Ileostomy #	NA
Weinstein et al. * [52] (2021)	USA	RCT	216 (IG: *n* = 106)	Adults (≥21)	NA	Multisite (Los Angeles, Philadelphia, New Haven); telehealth via Zoom	Usual care	During 4–5 weekly sessions and 6-month post-randomization	Cancer (*n* = 106, 100%)	Colostomy Ileostomy Urostomy	Permanent
Martín-Muñoz et al. [56] (2022)	Spain	RCT	52 (IG: *n* = 27)	Intervention: 58.9; Control: 60.48	Intervention: 10 (37.0%) Female; Control: 14 (54.2%) Female	Regional University Hospital of Malaga	Standard care	At hospital discharge (mean 10.08 days after surgery).	NA	Colostomy Ileostomy	Permanent Temporary
Krouse et al. * [49] (2024)	USA	RCT	191 (IG: *n* = 90)	Mean: 61.9 (Intervention), 66.7 (Usual Care)	Intervention: 49 (54.4%) Male, 41 (45.6%) Female; Usual Care: 54 (53.5%) Male, 47 (46.5%) Female	Three academic centres (UPenn, Yale, City of Hope)	Usual care	Baseline, post-intervention (approx. 5–6 weeks), and 6 months post-completion.	Cancer (*n* = 90, 100%)	Colostomy Ileostomy Urostomy	Permanent Temporary
Van Der Storm et al. [57] (2024)	Netherlands	RCT	208 (IG: *n* = 96)	Mean: 56	Intervention: 54 (56.3%) Male; Control: 53 (47.3%)	Two academic and 12 teaching hospitals	Restricted MHApp version	Baseline, two weeks, one month, and three months postoperatively	Cancer (*n* = 50, 52.1%) Non-cancer (*n* = 46, 47.9%)	Colostomy Ileostomy	NA
Wang et al. [55] (2024)	China	RCT	120 (IG: *n* = 60)	37–85 years (Mean: 51)	Male: 36 (60%), Female: 24 (40%)	Dept. of Anorectal Surgery, Hebei Medical University	Regular education and routine follow-up	Baseline (Day 0), 1 month, and 3 months	NA	Colostomy	Permanent
Maeng et al. * [50] (2025)	USA	RCT	204 (IG: *n* = 76)	Mean: 62.6 to 62.8	43.6% to 44.7% Female	Rural communities from eight participating sites	Usual care	Baseline, post-session (5 weeks), 3 months, and 6 months.	Cancer (*n* = 76, 100%)	Colostomy Ileostomy * Urostomy	Permanent Temporary
Sayar and Vural [45] (2025)	Turkey	NRCC	65 (IG: *n* = 31)	Intervention: 54.52 ± 15.29; Control: 53.67 ± 13.07	Intervention: 23 (74.2%) Male; Control: 29 (85.3%) Male	Stoma Therapy Unit, Dokuz Eylul University, Izmir	Routine care	Baseline, 3 months, and 6 months	Cancer (*n* = 21, 67.7%) Unknown (*n* = 10, 32.3%)	Colostomy Ileostomy	Permanent Temporary

* Publications arising from the Ostomy Self-Management Telehealth intervention; each publication addressed a distinct objective or dimension of the intervention. # or other small bowel stomas. Abbreviations: EARCT, Economic Analysis of an RCT; Exp: Experimental FPS, Feasibility pilot study; IG, Intervention Group; MHApp: Mobile health application; NA, Non-Available; NRCC: Non-Randomise Comparison Co-hort; PPC: Precourse-Postcourse Comparison; QE, Quasi-experimental; RCT, Randomised controlled trial; and USA, United States of America.

**Table 2:** Summary of features of peer-support interventions for self-management in adults living with an intestinal ostomy.

Study (Year)	Delivery and Administration	Format	Duration			Content		Resources	Technics/ Strategies	Intervention Leading	Part:Peer Ratio
			Inception	Session	Overall Duration	Developers	Description Summary				
Cheng et al. [44]	Face-to-face Group	1 sess/wk	Post-op: 8 mo–6 y	120 min	3 wk	RN ON	Stoma care: changing bags, complication prevention, diet and daily activities Positive SM	(1) Trained peers (2) SM handbook (3) Lectures	(a) Structured content (b) Experience-sharing (c) Group education	Peer	13–14:2
Altuntas et al. [53]	Face-to-face Group	1 sess	Post-op: 1–75 mo	450 min	1 day	PHE ON Surgeon	Stoma definition and need Stoma care and complications Social gathering	(3) Lectures (4) Video (5) Brochures (6) Stomatherapy unit phone numbers	(a) Structured content (d) Q&A sess (b) Experience-sharing (c) Group education	Surgeon and ON	NA
Karabulut et al. [54] (2014)	Face-to-face Group	1 sess/wk	Post-op: 13–60 mo	90 min	6 wk	ON	Physiological problems and daily life Psychological problems Sex life, relationships, communication Social activity Future life plans	(3) Lectures (7) Slide presentations (8) Stoma model and materials	(a) Structured content (e) Planned group interaction (f) Demonstration (g) Discussion (d) Q&A sess (b) Experience-sharing (c) Group education	ON	7–10:7–10
Krouse et al. * [48] (2016)	Face-to-face Group	2–3 sess/day #: Day 1 sess 1 and 2 Day 30 sess 3, 4 and 5	Pre-op (NA) to Post-op: 22–1626 day	60 min	1 mo	RN ON Surgeon	SM and immediate concerns Social well-being and body image Healthy lifestyle	(1) Trained peers (9) Matched peer: gender (10) Hands-on laboratory (11) DVD (12) Other visual content (13) Supplemental peer contact	(h) Adults teaching principles (a) Structured content (i) Interactive teaching (g) Discussion (j) Role-playing (k) Problem-oriented approach (b) Experience-sharing (c) Group education	Peer and ON	4:2
Hornbrook et al. * [47]	Face-to-face Group	2–3 sess/day #: Day 1 sess 1 and 2 Day 30 sess 3, 4 and 5	Pre-op (NA) to Post-op: 22–1626 day	60 min	1 mo	RN ON Surgeon	SM and immediate concerns Social well-being and body image Healthy lifestyle	(1) Trained peers (9) Matched peer: gender (10) Hands-on laboratory (11) DVD (12) Other visual content (13) Supplemental peer contact	(h) Adults teaching principles (a) Structured content (g) Discussion (j) Role-playing (k) Problem-oriented approach (b) Experience-sharing (c) Group education	Peer and ON	4:2
Sun et al. * [51] (2018)	Remote Video conf Group	2–3 sess/day #: Day 1 sess 1 and 2 Day 30 sess 3	Post-op: NA	60 min	1 mo	RN ON Surgeon	SM and immediate concerns Social well-being and body image Healthy lifestyle	(14) Video conf (15) Technical support (16) Reminder calls (1) Trained peers (9) Matched peer: gender (17) Workbook (7) Slide presentations (18) Diagrams (19) Photos (20) Close-ups views (21) Site resource list	(h) Adults teaching principles (a) Structured content (i) Interactive teaching (g) Discussion (j) Role-playing (k) Problem-oriented approach (b) Experience-sharing (l) Distance learning teaching (c) Group education	Peer and ON	6:2
Fearn et al. [46] (2020)	Remote MHApp Individual	Continuous	Pre-op (NA) to Post-op (≤day 4): NA	NA	1 mo	NA	Biometrics transmission: ostomy output, device usage, skin condition Health education: NA Early complication intervention	(1) Trained peers (22) MHApp: stoma device connected (23) HT monitoring (24) Continuous HT link (25) Notifications: clinical	(m) Digital health education (b) Experience-sharing (n) Self-learning education	Peer assisted	NA
Weinstein et al. * [52] (2021)	Remote Video conf Group	1 sess/wk #	Post-op: NA	90–120 min	1 mo	RN ON Surgeon	SM and immediate concerns Social well-being and body image Healthy lifestyle	(14) Video conf (15) Technical support (16) Reminder calls (17) Workbook (1) Trained peers (9) Matched peer: gender (18) Diagrams (19) Photos (20) Close-ups views (21) Site resource list	(a) Structured content (g) Discussion (j) Role-playing (h) Adults teaching principles (k) Problem-oriented approach (b) Experience-sharing (l) Distance learning teaching (c) Group education	Peer and ON	4–6:4–6
Martín-Muñoz et al. [56] (2022)	Face-to-face Individual Visit	1 visit	Post-op: afternoon before discharge	NA	NA	NA	Peer visit: participants listening, emotional support, sharing experiences, positive example	(1) Trained peers (9) Matched-peer: gender and age	(o) Active listening (b) Experience-sharing	Peer	1:1
Krouse et al. * [49] (2024)	Remote Video conf Group	1 sess/wk #	Post-op: NA	90 min	5–6 wk	RN ON Surgeon	SM and immediate concerns Social well-being and body image Healthy lifestyle	(14) Video conf (15) Technical support (16) Reminder calls (17) Workbook (1) Trained peers (9) Matched peer: gender (18) Diagrams (19) Photos (20) Close-ups views (21) Site resource list	(a) Structured content (g) Discussion (j) Role-playing (h) Adults teaching principles (k) Problem-oriented approach (b) Experience-sharing (l) Distance learning teaching (c) Group education	Peer and ON	4–6:4–6
Van Der Storm et al. [57] (2024)	Remote MHApp Individual	Continuous	Pre-op (≥day 21) to Post-op (≤day 5): NA	NA	3 mo	Health providers Stoma patients	Info: associations, library, stoma Clinical pathway timeline Patient clinical process Stoma output registration Peer-support platform Peer chat	(22) MHApp (4) Video (26) Illustration (25) Notifications: personalised time-guided (27) Peer selection (28) Chat	(m) Digital health education (b) Experience-sharing (n) Self-learning education	Peer assisted	1:1
Wang et al. [55] (2024)	Hybrid: Face-to-face Home visit Remote Telephone WeChat Individual Face-to-face Group	VHE Continuous Peer Education 1 consult/wk 1 meeting/mo	Post-op from discharge: NA	Individual sess NA Group sess 60–120 min	3 mo	Peer Psychologist Nutritionist Researchers ON	VHE: environment intro, disease intro, successful cases Peer education: NA	(1) Trained peers (4) Video (29) Posters (5) Brochures (12) Other visual content (30) Telephone (31) WeChat (32) Home visit	(p) Personalised educational content (b) Experience-sharing (q) Combined individual and group education	Peer and ON	10:2
Maeng et al. * [50] (2025)	Remote Video conf Group	1 sess/wk #	Post-op: NA	90 min	5 wk	RN ON Surgeon	SM and immediate concerns Social well-being and body image Healthy lifestyle	(14) Video conf (15) Technical support (16) Reminder calls (17) Workbook (1) Trained peers (9) Matched peer: gender (18) Diagrams (19) Photos (20) Close-ups views (21) Site resource list	(a) Structured content (g) Discussion (j) Role-playing (h) Adults teaching principles (k) Problem-oriented approach (b) Experience-sharing (l) Distance learning teaching (c) Group education	Peer and ON	4–6:4–6
Sayar and Vural [45] (2025)	Face-to-face Group	1 sess/mo	Post-op: 2–6 mo	90–120 min	4 mo	RN Peers	Stoma care Coping strategies sharing to deal with an ostomy complications Experience and difficulties sharing on stoma care Experience and problem sharing on nutrition, exercise or social life	(4) Video	(e) Planned group interaction (p) Personalized educational content (b) Experience-sharing (c) Group education (g) Discussion (d) Q&A sess	RN	9–12:9–12

* Publications arising from the Ostomy Self-Management Telehealth intervention; each publication addressed a distinct objective or dimension of the intervention. # Three mandatory sessions of a total-of-five-sessions curriculum. Abbreviations: Consult, consultation; HT, health team; Info, information; Intro, introduction; MHApp, mobile healthcare application; mo, month; NA, non-available; ON, ostomy nurse, stomatherapy nurse or enterostomal therapist or wound, ostomy and continence nurse or expert stoma care nurse; Part, participant; Post-op, post-operative; Pre-op, pre-operative; PHE, public health expert; Q&A, question and answer; RN, research nurse; sess, session; SM, self-management; VHE, visual health education; Video conf, video conference; wk, week; and y, year.

**Table 3:** Implementation facilitators and barriers to peer-support interventions for self-management in adults living with an intestinal ostomy.

Domain	Implementation Facilitators	Implementation Barriers
Education and communication	Experiential knowledge-sharing supporting communication and learning [44,45]; problem-solving learning strategies supporting self-management and participation [54]; peer-led education facilitating knowledge transfer and reducing communication barriers between nurses and patients [44,53,54,55,57]	Nr
Patient engagement and support	Active patient involvement in meeting organisation and peer interaction [53,55,57]; incorporation of patients’ identified needs and preferred functionalities into programme design [53,55,57]; tailoring of educational content to participants’ needs and preferences [45,57]; collaboration between ostomy nurses, peers, and patient associations supporting programme delivery and engagement [44,55,57]	Cultural beliefs, stigma, and societal taboos surrounding sensitive topics, particularly sexual health, limiting participation [44]
Accessibility and delivery	Remote delivery facilitating participation and access to peer support, particularly for geographically dispersed or rural participants [46,49,50,51,52,55,57]	Travel distance, transportation difficulties, physical limitations, and financial constraints limiting participation in face-to-face interventions [47,48,49,50,51]
Technical and organisational factors	Digital platforms enabling remote access to peer support [46,49,50,51,52,55,57]	Development and maintenance costs of mobile healthcare applications [57]; delayed or inadequate responses to technical problems [57]; need for organisational and financial resources to maintain digital delivery [57]

Abbreviations: Nr, non-reported.

## Data Availability

Data extracted and synthesised for this review are available in the article. Further inquiries can be directed to the corresponding author.

## Supplementary Materials

The following supporting information can be downloaded at: https://www.mdpi.com/article/10.3390/nursrep16090325/s1, File S1: Preferred Reporting Items for Systematic reviews and Meta-Analyses extension for Scoping Reviews (PRISMA-ScR) Checklist; Table S1: Outcome data from included publications.

## Public Involvement Statement

No public involvement in any aspect of this research.

## Guidelines and Standards Statement

This manuscript was prepared in accordance with the Preferred Reporting Items for Systematic Reviews and Meta-Analyses Extension for Scoping Reviews (PRISMA-ScR).

## Abbreviations

The following abbreviations are used in this manuscript:

PRISMA	Preferred Reporting Items for Systematic reviews and Meta-Analyses
PRISMA-ScR	Preferred Reporting Items for Systematic reviews and Meta-Analyses extension for Scoping Reviews
PSI	Peer-support intervention

## Appendix A

**Table A1:** Complete electronic database search strategies organised by review concepts.

Database	Population (Intestinal Ostomy)	Concept 1 (Peer Support)	Concept 2 (Self-Management)
MEDLINE (PubMed)	Colostomy [MeSH Terms] OR Enterostomy [MeSH Terms] OR Ileostomy [MeSH Terms] OR Ostomy [MeSH Terms] OR colostom* [Title/Abstract] OR enterostom* [Title/Abstract] OR ileostom* [Title/Abstract] OR ostom* [Title/Abstract] OR stoma* [Title/Abstract]	Patient Education as Topic [MeSH Terms] OR Peer Group [MeSH Terms] OR Self-help Groups [MeSH Terms] OR Social Support [MeSH Terms] OR Patient Navigation [MeSH Terms] OR coach* [Title/Abstract] OR health advisor [Title/Abstract] OR lay-led [Title/Abstract] OR laymen [Title/Abstract] OR lived experience [Title/Abstract] OR mentor* [Title/Abstract] OR lay worker [Title/Abstract] OR navigat* [Title/Abstract] OR peer support [Title/Abstract] OR peer-support [Title/Abstract] OR peer to peer [Title/Abstract]	Quality of Life [MeSH Terms] OR Self Efficacy [MeSH Terms] OR Self-Care [MeSH Terms] OR Self-Management [MeSH Terms] OR empowerment [Title/Abstract] OR quality of life [Title/Abstract] OR selfcare [Title/Abstract] OR self-care [Title/Abstract] OR selfefficacy [Title/Abstract] OR self-efficacy [Title/Abstract] OR selfmanagement [Title/Abstract] OR self-management [Title/Abstract] OR selfmonitor* [Title/Abstract] OR self-monitor* [Title/Abstract]
CINAHL (EBSCOhost)	MH “Colostomy” OR MH “Enterostomy” OR MH “Ileostomy” OR MH “Ostomy” OR TI colostom* OR AB colostom* OR TI enterostom* OR AB enterostom* OR TI ileostom* OR AB ileostom* OR TI ostom* OR AB ostom* OR TI stoma* OR AB stoma*	MH “Patient Education” OR MH “Patient Navigation” OR MH “Peer Group” OR MH “Support Groups” OR MH “Support, Social” OR TI coach* OR AB coach* OR TI “health advisor” OR AB “health advisor” OR TI “lay health worker*” OR AB “lay health worker*” OR TI “lay worker” OR AB “lay worker” OR TI lay-led OR AB lay-led OR TI laymen OR AB laymen OR TI “lived experience” OR AB “lived experience” OR TI mentor* OR AB mentor* OR TI navigat* OR AB navigat* OR TI “peer support” OR AB “peer support” OR TI peer-support OR AB peer-support OR TI “peer to peer” OR AB “peer to peer” OR TI peer* OR AB peer* OR TI peer-led OR AB peer-led OR TI “psychosocial support” OR AB “psychosocial support” OR TI self-help OR AB self-help OR TI “social support” OR AB “social support” OR TI “support group*” OR AB “support group*” OR TI “support intervention*” OR AB “support intervention*”	MH “Quality of Life” OR MH “Self-Efficacy” OR MH “Self Care” OR MH “Self-Management” OR TI empowerment OR AB empowerment OR TI “quality of life” OR AB “quality of life” OR TI selfcare OR AB selfcare OR TI self-care OR AB self-care OR TI selfefficacy OR AB selfefficacy OR TI self-efficacy OR AB self-efficacy OR TI selfmanagement OR AB selfmanagement OR TI self-management OR AB self-management OR TI selfmonitor* OR AB selfmonitor* OR TI self-monitor* OR AB self-monitor*
APA PsycINFO (ProQuest)	noft(colostom*) OR noft(enterostom*) OR noft(ileostom*) OR noft(ostom*) OR noft(stoma*)	noft(“health advisor”) OR noft(“lay health worker*”) OR noft(“lay worker*”) OR noft(lay-led) OR noft(laymen) OR noft(“lived experience”) OR noft(mentor*) OR noft(coach*) OR noft(navigat*) OR noft(“patient education”) OR noft(“patient navigation”) OR noft(“peer group”) OR noft(“peer support”) OR noft(“peer to peer”) OR noft(peer*) OR noft(peer-led) OR noft(“psychosocial support”) OR noft(self-help) OR noft(“social support”) OR noft(“support group*”) OR noft(“support intervention*”)	noft(empowerment) OR noft(“quality of life”) OR noft(“self-care”) OR noft(“self-efficacy”) OR noft(“self-management”) OR noft(self-monitor*)

AB, abstract; MH, CINAHL Subject Heading; MeSH, Medical Subject Headings; noft, anywhere except full text; TI, title. Search strategies were developed for MEDLINE (PubMed) and subsequently adapted to the controlled vocabulary, indexing terms, field codes, and search functionality of each database. Boolean operators (AND, OR) were used to combine the Population, Concept, and Context concepts within each database.

## References

[1] Juma I.M., Qassim T., Saeed M.F., Qassim A., Al-Rawi S., Al-Salmi S., Salman M.T., Al-Saadi I., Almutawea A., Aljahmi E. (2023). Intestinal Stomas—Current Practice and Challenges: An Institutional Review. Euroasian J. Hepato-Gastroenterol..

[2] Carlsson E., Forsmark A., Sternhufvud C., Scheffel G., Andersen F.B., Persson E.I. (2023). Short- and Long-Term Direct and Indirect Costs of Illness after Ostomy Creation—A Swedish Nationwide Registry Study. BMC Health Serv. Res..

[3] Massenga A., Chibwae A., Nuri A.A., Bugimbi M., Munisi Y.K., Mfinanga R., Chalya P.L. (2019). Indications for and Complications of Intestinal Stomas in the Children and Adults at a Tertiary Care Hospital in a Resource-Limited Setting: A Tanzanian Experience. BMC Gastroenterol..

[4] Goodman W., Downing A., Allsop M., Munro J., Taylor C., Hubbard G., Beeken R.J. (2022). Quality of Life Profiles and Their Association with Clinical and Demographic Characteristics and Physical Activity in People with a Stoma: A Latent Profile Analysis. Qual. Life Res..

[5] Poliani A., Marcomini I., Butti P., Nedesca E.D., Manara D.F., Villa G. (2026). Stoma Leakage: Prevalence, Associated Factors, and Assessment Tools—A Scoping Review. Nurs. Rep..

[6] Ayik C., Özden D., Cenan D. (2020). Ostomy Complications, Risk Factors, and Applied Nursing Care: A Retrospective, Descriptive Study. Wound Manag. Prev..

[7] Burgess-Stocks J., Gleba J., Lawrence K., Mueller S. (2022). Ostomy and Continent Diversion Patient Bill of Rights: Research Validation of Standards of Care. J. Wound Ostomy Cont. Nurs..

[8] Claessens I., Probert R., Tielemans C., Steen A., Nilsson C., Andersen B.D., Størling Z.M. (2015). The Ostomy Life Study: The Everyday Challenges Faced by People Living with a Stoma in a Snapshot. Gastrointest. Nurs..

[9] Registered Nurses’ Association of Ontario (2019). Supporting Adults Who Anticipate or Live with an Ostomy.

[10] Goldberg M., Colwell J., Burns S., Carmel J., Fellows J., Hendren S., Livingston V., Nottingham C.U., Pittman J., Rafferty J. (2018). WOCN Society Clinical Guideline: Management of the Adult Patient with a Fecal or Urinary Ostomy—An Executive Summary. J. Wound Ostomy Cont. Nurs..

[11] Lorig K.R., Holman H.R. (2003). Self-Management Education: History, Definition, Outcomes, and Mechanisms. Ann. Behav. Med..

[12] Grant M., McCorkle R., Hornbrook M.C., Wendel C.S., Krouse R. (2013). Development of a Chronic Care Ostomy Self-Management Program. J. Cancer Educ..

[13] Sarmiento L., González G. (2025). Experiencia de Automanejo En Adultos Con Colostomías Por Cáncer Colorrectal. Rev. Cuid..

[14] Grady P.A., Gough L.L. (2014). Self-Management: A Comprehensive Approach to Management of Chronic Conditions. Am. J. Public Health.

[15] Chabal L.O., Prentice J.L., Ayello E.A. (2021). Practice Implications from the WCET^®^ International Ostomy Guideline 2020. Adv. Ski. Wound Care.

[16] Zelga P., Kluska P., Zelga M., Piasecka-Zelga J., Dziki A. (2021). Patient-Related Factors Associated with Stoma and Peristomal Complications Following Fecal Ostomy Surgery: A Scoping Review. J. Wound Ostomy Cont. Nurs..

[17] Bozkul G., Senol Celik S., Nur Arslan H. (2024). Nursing Interventions for the Self-Efficacy of Ostomy Patients: A Systematic Review. J. Tissue Viability.

[18] Faury S., Koleck M., Foucaud J., M’Bailara K., Quintard B. (2017). Patient Education Interventions for Colorectal Cancer Patients with Stoma: A Systematic Review. Patient Educ. Couns..

[19] Mohamed N.E., Shah Q.N., Kata H.E., Sfakianos J., Given B. (2021). Dealing with the Unthinkable: Bladder and Colorectal Cancer Patients’ and Informal Caregivers’ Unmet Needs and Challenges in Life After Ostomies. Semin. Oncol. Nurs..

[20] Andersen F.B., Kjellberg J., Ibsen R., Sternhufvud C., Petersen B. (2024). The Clinical and Economic Burden of Illness in the First Two Years after Ostomy Creation: A Nationwide Danish Cohort Study. Expert Rev. Pharmacoeconomics Outcomes Res..

[21] Maeng D., Hoffman R.L., Sun V., Sticca R.P., Krouse R.S. (2025). Post-Surgical Acute Care Utilization and Cost of Care among Cancer Survivors with an Ostomy: Findings from Three Large Hospital Systems in the United States. J. Cancer Policy.

[22] Thompson D.M., Booth L., Moore D., Mathers J. (2022). Peer Support for People with Chronic Conditions: A Systematic Review of Reviews. BMC Health Serv. Res..

[23] Price A., De Bell S., Shaw N., Bethel A., Anderson R., Coon J.T. (2022). What Is the Volume, Diversity and Nature of Recent, Robust Evidence for the Use of Peer Support in Health and Social Care? An Evidence and Gap Map. Campbell Syst. Rev..

[24] Hossain S.N., Jaglal S.B., Shepherd J., Perrier L., Tomasone J.R., Sweet S.N., Luong D., Allin S., Nelson M.L.A., Guilcher S.J.T. (2021). Web-Based Peer Support Interventions for Adults Living with Chronic Conditions: Scoping Review. JMIR Rehabil. Assist. Technol..

[25] Verma I., Gopaldasani V., Jain V., Chauhan S., Chawla R., Verma P.K., Hosseinzadeh H. (2022). The Impact of Peer Coach-Led Type 2 Diabetes Mellitus Interventions on Glycaemic Control and Self-Management Outcomes: A Systematic Review and Meta-Analysis. Prim. Care Diabetes.

[26] Byfield D. (2020). The Lived Experiences of Persons with Ostomies Attending a Support Group: A Qualitative Study. J. Wound Ostomy Cont. Nurs..

[27] Ding J.-Y., Pan T.-T., Lu X.-J., You X.-M., Qi J.-X. (2024). Effects of Peer-Led Education on Knowledge, Attitudes, Practices of Stoma Care, and Quality of Life in Bladder Cancer Patients after Permanent Ostomy. Front. Med..

[28] Adriano A., Thompson D.M., McMullan C., Price M., Moore D., Booth L., Mathers J. (2022). Peer Support for Carers and Patients with Inflammatory Bowel Disease: A Systematic Review. Syst. Rev..

[29] Hu J., Wang X., Guo S., Chen F., Wu Y., Ji F., Fang X. (2019). Peer Support Interventions for Breast Cancer Patients: A Systematic Review. Breast Cancer Res. Treat..

[30] Levy B.B., Luong D., Perrier L., Bayley M.T., Munce S.E.P. (2019). Peer Support Interventions for Individuals with Acquired Brain Injury, Cerebral Palsy, and Spina Bifida: A Systematic Review. BMC Health Serv. Res..

[31] Aibibula M., Burry G., Gagen H., Osborne W., Lewis H., Bramwell C., Pixley H., Cinque G. (2022). Gaining Consensus: The Challenges of Living with a Stoma and the Impact of Stoma Leakage. Br. J. Nurs..

[32] Islam K.F., Awal A., Mazumder H., Munni U.R., Majumder K., Afroz K., Tabassum M.N., Hossain M.M. (2023). Social Cognitive Theory-Based Health Promotion in Primary Care Practice: A Scoping Review. Heliyon.

[33] De La Fuente J., Kauffman D.F., Boruchovitch E. (2023). Editorial: Past, Present and Future Contributions from the Social Cognitive Theory (Albert Bandura). Front. Psychol..

[34] Liblub S., Pringle K., McLaughlin K., Cummins A. (2024). Peer Support and Mobile Health for Perinatal Mental Health: A Scoping Review. Birth.

[35] Grant E., Johnson L., Prodromidis A., Giannoudis P.V. (2021). The Impact of Peer Support on Patient Outcomes in Adults with Physical Health Conditions: A Scoping Review. Cureus.

[36] Ziegler E., Hill J., Lieske B., Klein J., Dem O.V., Kofahl C. (2022). Empowerment in Cancer Patients: Does Peer Support Make a Difference? A Systematic Review. Psycho-Oncology.

[37] Heydari A., Manzari Z., Pouresmail Z. (2023). Nursing Intervention for Quality of Life in Patients with Ostomy: A Systematic Review. Iran. J. Nurs. Midwifery Res..

[38] Goodman W., Allsop M., Downing A., Munro J., Taylor C., Hubbard G., Beeken R.J. (2022). A Systematic Review and Meta-analysis of the Effectiveness of Self-management Interventions in People with a Stoma. J. Adv. Nurs..

[39] Peters M.D., Godfrey C., McInerney P., Munn Z., Tricco A.C., Khalil H. (2024). Scoping Reviews (2020). JBI Manual for Evidence Synthesis.

[40] Tricco A.C., Lillie E., Zarin W., O’Brien K.K., Colquhoun H., Levac D., Moher D., Peters M.D.J., Horsley T., Weeks L. (2018). PRISMA Extension for Scoping Reviews (PRISMA-ScR): Checklist and Explanation. Ann. Intern. Med..

[41] Dennis C.-L. (2003). Peer Support within a Health Care Context: A Concept Analysis. Int. J. Nurs. Stud..

[42] Simoni J.M., Franks J.C., Lehavot K., Yard S.S. (2011). Peer Interventions to Promote Health: Conceptual Considerations. Am. J. Orthopsychiatry.

[43] Page M.J., McKenzie J.E., Bossuyt P.M., Boutron I., Hoffmann T.C., Mulrow C.D., Shamseer L., Tetzlaff J.M., Akl E.A., Brennan S.E. (2021). The PRISMA 2020 Statement: An Updated Guideline for Reporting Systematic Reviews. BMJ.

[44] Cheng F., Xu Q., Dai X., Yang L. (2012). Evaluation of the Expert Patient Program in a Chinese Population with Permanent Colostomy. Cancer Nurs..

[45] Sayar S., Vural F. (2025). Effects of a Support Group Intervention on Adaptation to Ostomy, Quality of Life, and Complication Severity in Patients with an Ostomy. J. Wound Ostomy Cont. Nurs..

[46] Fearn R.I., Gorgun E., Sapci I., Mehta S.N., Dinh B., Yowell Q.V., Eisenstein S. (2020). Improved 30-Day Surgical Outcomes in Ostomates Using a Remote Monitoring and Care Management Program: An Observational Study. Dis. Colon Rectum.

[47] Hornbrook M.C., Cobb M.D., Tallman N.J., Colwell J., McCorkle R., Ercolano E., Grant M., Sun V., Wendel C.S., Hibbard J.H. (2018). Costs of an Ostomy Self-management Training Program for Cancer Survivors. Psycho-Oncology.

[48] Krouse R.S., Grant M., McCorkle R., Wendel C.S., Cobb M.D., Tallman N.J., Ercolano E., Sun V., Hibbard J.H., Hornbrook M.C. (2016). A Chronic Care Ostomy Self-management Program for Cancer Survivors. Psycho-Oncology.

[49] Krouse R.S., Zhang S., Wendel C.S., Sun V., Grant M., Ercolano E., Hornbrook M.C., Cidav Z., Nehemiah A., Rock M. (2024). A Randomized Prospective Trial of an Ostomy Telehealth Intervention for Cancer Survivors. Cancer.

[50] Maeng D., Sun V., Nielsen M.E., Hoffman R.L., Garg T., Popek S.M., Sticca R.P., Aka A.A., Hesham W.M., Grant M. (2025). Impact of Ostomy Self-Management Telehealth Training for Rural Cancer Survivors on Health Care Utilization and Economic Outcomes in the United States. J. Rural Health.

[51] Sun V., Ercolano E., McCorkle R., Grant M., Wendel C.S., Tallman N.J., Passero F., Raza S., Cidav Z., Holcomb M. (2018). Ostomy Telehealth for Cancer Survivors: Design of the Ostomy Self-Management Training (OSMT) Randomized Trial. Contemp. Clin. Trials.

[52] Weinstein R.S., Holcomb M.J., Mo J., Yonsetto P., Bojorquez O., Grant M., Wendel C.S., Tallman N.J., Ercolano E., Cidav Z. (2021). An Ostomy Self-Management Telehealth Intervention for Cancer Survivors: Technology-Related Findings from a Randomized Controlled Trial. J. Med. Internet Res..

[53] Altuntas Y.E., Kement M., Gezen C., Eker H.H., Aydin H., Sahin F., Okkabaz N., Oncel M. (2012). The Role of Group Education on Quality of Life in Patients with a Stoma. Eur. J. Cancer Care.

[54] Karabulut H.K., Dinç L., Karadag A. (2014). Effects of Planned Group Interactions on the Social Adaptation of Individuals with an Intestinal Stoma: A Quantitative Study. J. Clin. Nurs..

[55] Wang Y., Ren H., Li M., Xie L., Lin L., Fang Y.-L. (2024). Effect of Enterostomal Therapist-Led Visual Health Education Combined with Peer Education on the Self-Nursing Ability, Quality of Life and Peristomial Complications in Patients with a Permanent Colostomy. Patient Prefer. Adherence.

[56] Martín-Muñoz B., Montesinos-Gálvez A.C., Crespillo-Díaz A.Y., Jódar-Sánchez F. (2022). Efficacy of a Social Interaction Intervention in Early Postoperative Period to Improve Coping in Persons with an Ostomy: A Randomized Controlled Trial. J. Wound Ostomy Cont. Nurs..

[57] Van Der Storm S.L., Consten E.C.J., Govaert M.J.P.M., Tuynman J.B., Oosterling S.J., Grotenhuis B.A., Smits A.B., Marsman H.A., Van Rossem C.C., Van Duyn E.B. (2024). Better Stoma Care Using the Stoma App: Does It Help? A First Randomized Double-Blind Clinical Trial on the Effect of Mobile Healthcare on Quality of Life in Stoma Patients. Surg. Endosc..

[58] Pienaar M., Reid M. (2020). Self-Management in Face-to-Face Peer Support for Adults with Type 2 Diabetes Living in Low- or Middle-Income Countries: A Systematic Review. BMC Public Health.

[59] Hoey L.M., Ieropoli S.C., White V.M., Jefford M. (2008). Systematic Review of Peer-Support Programs for People with Cancer. Patient Educ. Couns..

[60] Haesler E., Ayello E.A., Chabal L.O., Hibbert D., Prentice J.L., Pereira J.C., Nunoo-Mensah J.W., Atkinson J., McNair R. (2025). Methodology for the World Council of Enterostomal Therapists^®^ International Ostomy Guideline Third Edition. World Counc. Enteros. Ther..

[61] Hui-Ren Z., Jin Z., Pian Z., Wei-ying Z. (2025). Applications of Telemedicine in Patients with an Ostomy: A Scoping Review. Adv. Ski. Wound Care.

[62] Soares-Pinto I., Braga A.M.P., Santos I.M.R.M.A., Ferreira N.M.R.G., Silva S.C.D.R.E., Alves P.J. (2023). eHealth Promoting Stoma Self-Care for People with an Elimination Ostomy: Focus Group Study. JMIR Hum. Factors.

[63] Soares-Pinto I.E., Queirós S.M.M., Alves P.J.P., Santos C.S.V.d.B., de Brito M.A.C. (2022). Promotion of Bowel Elimination Ostomy Self-Care: A Qualitative Study Based on the Nurses’ and Patients’ Perspectives. Nurs. Pract. Today.

[64] Teixeira C., Reis S. (2025). Benefits of Patient and Public Involvement in Cancer Research: Literature Review. Onco. News.

[65] Elliott M.J., Donald M., Farragher J., Verdin N., Love S., Manns K., Baragar B., Sparkes D., Fox D., Hemmelgarn B.R. (2023). Priorities for Peer Support Delivery among Adults Living with Chronic Kidney Disease: A Patient-Oriented Consensus Workshop. CMAJ Open.

[66] Thompson J., Bissell P., Cooper C.L., Armitage C.J., Barber R. (2014). Exploring the Impact of Patient and Public Involvement in a Cancer Research Setting. Qual. Health Res..

[67] Essex R., Booth L., Sirois F., Burch J., Dibley L. (2025). A Scoping Review of the Qualitative Literature Reporting Experiences of Living with a Stoma for Inflammatory Bowel Disease. J. Adv. Nurs..

[68] Yannitsos D., Murphy R.A., Pollock P., Di Sebastiano K.M. (2020). Facilitators and Barriers to Participation in Lifestyle Modification for Men with Prostate Cancer: A Scoping Review. Eur. J. Cancer Care.

