# Supplementary material for: Peer-Support Interventions for Self-Management in Adults Living with an Intestinal Ostomy: A Scoping Review
Source: Nurs Rep. 2026 Sep 6;16(9):325. doi: 10.3390/nursrep16090325 (PMC13610237; doi:10.3390/nursrep16090325)
Supplement: Supplementary file 1 [file nursrep-16-00325-s001.zip › Table S1_Outcomes_05.09.2026.pdf]

Table S1. Outcome data from included publications

Study (year)	Primary vs secondary outcomes	Main reported outcome	Measurement instruments	Statistical significance	Null and contradictory findings
Cheng et al. [44] (2012)	Primary: Knowledge, self-efficacy, self-management, and psychosocial adjustment. Secondary: Participant satisfaction.	Significant improvement in stoma knowledge, self-efficacy, self-management, and psychosocial adjustment.	Stoma-Related Knowledge Scale; Stoma Care Self-efficacy Scale; Self-management Scale; Psychosocial Adjustment Scale.	Total scores for knowledge, self-efficacy, self-management, and psychosocial adjustment all showed ($p < 0.01$).	No significant improvement in self-efficacy regarding social life ($p = 0.113$), sexual life ($p=0.430$), or keeping energy ($p = 0.095$).
Altuntas et al. [53] (2012)	Primary: Quality of Life improvement. Secondary: Predictive demographic factors for improvement.	Significant improvement in 7 out of 8 Short Form-36 (SF-36) scale profiles and mental health summary measures 3 months after group education.	SF-36	Significant improvement ($p <0.05$) in Physical Functioning, Bodily Pain, Social Functioning, Role-Emotional, Mental Health, and Physical Health/Mental Health	No statistically significant improvement was found in the Vitality scale ($p = 0.159$).
Karabulut et al. [54] (2014)	Primary: Effects of planned group interaction on social adjustment. Secondary: Psychosocial adjustment to illness and socio-demographic/stoma attributes.	Planned group interaction significantly increased Ostomy Adjustment Inventory (OAI-23) scores and improved psychosocial adjustment scores in the experimental group.	OAI-23; Psychosocial Adjustment to Illness Scale-Self-Report (PAIS-SR); Socio-demographic questionnaire.	$p < 0.05$ for improvements in OAI and PAIS-SR scores in the experimental group; no significant change ($p > 0.05$) in the control group.	No significant changes occurred in the control group over time. Working individuals or those with temporary stomas sometimes faced unique adaptation challenges or lower initial scores.
Krouse et al.* [48] (2016)	Primary: Patient Activation, self-efficacy, and Health-Related Quality of Life (HRQOL). Secondary: Anxiety, depression, and care satisfaction.	Significant improvements observed in patient activation ($p < 0.0001$), total HRQOL ($p = 0.01$), and social well-being ($p = 0.002$).	Patient Activation Measure (PAM), Self-efficacy scale, City of Hope Quality of Life Ostomy (COH-QOL-O), Hospital Anxiety and Depression Scale (HADS), Group Health Association	PAM ($p < 0.0001$), total HRQOL ($p = 0.02$), social well-being ($p = 0.002$), and follow-up anxiety ($p = 0.02$) were significant.	Self-efficacy ($p = 0.07$) and psychological/spiritual well-being did not show significant improvement. Depression showed only a trend ($p = 0.09$).

			of America Satisfaction with Ostomy Care Survey.		
Hornbrook et al.* [47] (2018)	Primary: Incremental expenses to surgical practice for delivery. Secondary: HRQOL, patient activation, self-efficacy, and anxiety.	Total incremental expense per cohort of four survivors was 7,246$, or 1,812 per patient.	Labor and non-labor cost tracking (referencing PAM, Self-efficacy scale, and COH-QOL-O scales from associated study).	Significant improvement in PAM (p = 0.0004), SE (p=0.006), HRQOL (p = 0.01), and reduced anxiety (p = 0.047).	Nine participants dropped out due to death, illness, or travel distance. Lack of technology familiarity was a barrier to participation.
Sun et al*. [51] (2018)	Primary: Survivor activation, self-efficacy, ostomy-related knowledge, and HRQOL. Secondary: Medical care utilization and family financial burden.	Pilot study results showed significant improvement in patient activation, self-efficacy, total HRQOL, and physical/social well-being.	PAM, Self-Efficacy Scale, COH-QOL-O, Ostomy Knowledge Questionnaire, HADS, Burden of Ostomy Care Tool.	Pilot: PAM (p = 0.0004), self-efficacy (p = 0.006), total HRQOL (p=0.01), physical (p=0.005), and social well-being (p = 0.002).	Pilot study showed no significant improvement in psychological well-being (p = 0.14) or spiritual well-being (p = 0.64).
Fearn et al. [46] (2020)	Primary: Hospital-based acute care (HBAC) utilization (readmission, Emergency Department (ED) visit). Secondary: Patient characteristics and coach communication patterns.	HBAC complication rate of 21.2% (15.1% readmission, 6.1% ED visits) compared to historical data (38% HBAC).	Alfred: SmartBag (wireless sensors tracking output/leakage) and SmartCare platform.	Coach interactions (p = 0.024) and duration (p = 0.041) were significantly higher for HBAC users.	Sex, age, Body Mass Index, and procedure type did not significantly differ between groups (no statistical association with HBAC events).
Weinstein et al.* [52] (2021)	Primary: Telehealth technology–related (TTR) incidents and engineering support. Secondary: Participant acceptance, technology problems, and attrition.	97.1% of sessions were completed as scheduled; TTR incidents occurred in 36.3% of sessions but zero sessions were cancelled due to failure.	Field notes (nurse, peer, engineer), TTR incident categorization (major vs. minor), and Consolidated Standards of Reporting Trials flow data.	Not in source (Focus on descriptive frequencies/percentages).	7.4% of survivors declined due to technology concerns; 12.2% of the intervention arm opted out after randomization citing technology and illness.

Martín-Muñoz et al. [56] (2022)	Primary: Coping process. Secondary: Health beliefs and Acceptance of health status.	Intervention group achieved significantly higher mean coping process scores (3.90 vs 3.19, p = .002).	Nursing Outcomes Classification taxonomy (Coping, Health Beliefs, Acceptance of status) using 1-5 scale.	Coping (p = .002); Health beliefs (p = .0004); Acceptance of status (p = 0.0001).	Urgent ostomy surgery was negatively correlated with coping (β= −0.610; p = .005). No significant differences for temporary vs permanent ostomies.
Krouse et al.* [49] (2024)	Primary: Patient activation. Secondary: Self-efficacy, City of Hope quality of life-Ostomy, Hospital Anxiety and Depression Scale, HADS, and Ostomy Knowledge.	No clear overall benefit was seen for the OSMT intervention across the entire cohort, though indications of benefit were found for patients with urinary tumors and those with higher session attendance.	PAM, Self-efficacy, COH-QOL-O, HADS, and Ostomy Knowledge survey.	Nonsignificant improvement in PAM (p=0.72). Significant improvements (p < 0.05) in Self-efficacy and COH-O domains for urinary tumor and high-attendance subgroups.	No statistically significant difference between arms for the primary outcome in the total sample. Benefits in urinary tumors were not observed in colorectal or other tumor types.
Van der storm et al. [57] (2024)	Primary: Stoma-related quality of life. Secondary: Psychological adaptation, complications, readmittance, reoperations, and hospital stay.	A significant 3.1-point improvement in stoma-related quality of life at one month postoperatively after correction for confounding (p = 0.038).	Stoma Quality of Life scale, Karnofsky performance score, mobile proficiency, World Health Organization Quality of Life- Brief version (Physical, Psychological, Social, Environment), and disability score.	p = 0.038 for Stoma Quality of Life at 1 month; p = 0.047 for readmission rate; most secondary outcomes were not significant (p > 0.05).	No significant improvements for secondary outcomes like psychological adaptation. Intervention group had higher readmission rates (20.4% vs 10.0%) due to worse baseline Karnofsky scores.
Wang et al. [55] (2024)	Primary: Postoperative self-nursing ability, quality of life, and peristomial complications. Secondary: Adaptability, nursing satisfaction, mood, and daily living.	Improved quality of life (F = 342.734, p < 0.001), self-nursing ability (F = 256.321 , p < 0.001), and adaptability (F = 321.73, p < 0.001) in the study group.	Stoma Quality of Life scale; Exercise of Self-Care Agency; Ostomy Adjustment Scale (OAS); Satisfaction evaluation card; Complication tracking.	Quality of life, self-nursing, and adaptability all p < 0.001 at 3 months; complications and satisfaction were significant (p < 0.05).	No interaction effect found between treatment and time for total scores (p > 0.05). Failed to follow up long-term (beyond 3 months).

Maeng et al.* [50] (2025)	Primary: Health care utilization (nurse/physician visits, ED visits, acute care). Secondary: Economic outcomes (out-of-pocket (OOP) costs, employment, disability).	Lower frequencies of in-person nurse (-57.2%; p = 0.015) and physician (-76.1%; p = 0.024) visits in the post-session follow-up.	Surveys with mid-point calculations for interval-based value ranges (e.g., OOP cost scales).	Significant for nurse (p=0.015) and physician (p=0.024) visits at first follow-up; other measures (ED visits, costs, employment) were not significant (p > 0.05).	No significant differences in provider visits at 3 and 6-month follow-ups. ED visits, costs, and employment status failed to reach statistical significance.
Sayar and Vural [45] (2025)	Primary: Effect of support group on adaptation, HRQOL, and complication severity.	Intervention significantly improved quality of life, increased adaptation, and reduced complication severity.	COHQOL-O, OAS, and Pittman Ostomy Complication Severity Index (OCSI).	Significant differences in COHQOL-O (p = 0.016 at 6 mo), OAS (p = 0.005 at 6 months), and Pittman OCSI (p = 0.006 at 6 months).	No significant between-group difference found for Pittman OCSI scores at the first follow-up (p = 0.149). The control group showed no improvement over time.

*Publications arising from the Ostomy Self-Management Telehealth intervention; each publication addressed a distinct objective or dimension of the intervention.
